# Association Between Measurable Residual Disease in Patients With Intermediate-Risk Acute Myeloid Leukemia and First Remission, Treatment, and Outcomes

**DOI:** 10.1001/jamanetworkopen.2021.15991

**Published:** 2021-07-07

**Authors:** Sijian Yu, Zhiping Fan, Liping Ma, Yu Wang, Fen Huang, Qing Zhang, Jiafu Huang, Shunqing Wang, Na Xu, Li Xuan, Mujun Xiong, Lijie Han, Zhiqiang Sun, Hongyu Zhang, Hui Liu, Guopan Yu, Pengcheng Shi, Jun Xu, Meiqing Wu, Ziwen Guo, Yiying Xiong, Chongyang Duan, Jing Sun, Qifa Liu, Yu Zhang

**Affiliations:** 1 Department of Hematology, Nanfang Hospital, Southern Medical University, Guangzhou, China; 2 Department of Hematology, Sun Yat-Sen Memorial Hospital, Guangzhou, China; 3 Peking University People’s Hospital, Peking University Institute of Hematology, Beijing, China; 4 Department of Hematology, Guangdong Second Provincial General Hospital, Guangzhou, China; 5 Department of Hematology, Guangzhou First People’s Hospital, Guangzhou, China; 6 Department of Hematology, The First People’s Hospital of Chenzhou, Chenzhou, China; 7 Department of Hematology, The First Affiliated Hospital of Zhengzhou University, Zhengzhou, China; 8 Department of Hematology, Shenzhen Hospital of Southern Medical University, Shenzhen, China; 9 Department of Hematology, Shenzhen Hospital of Peking University, Shenzhen, China; 10 Department of Hematology, The First Affiliated Hospital of Guangxi Medical University, Guangxi, China; 11 Department of Hematology, Zhongshan People’s Hospital, Zhongshan, China; 12 Department of Hematology, The First Affiliated Hospital of Chongqing Medical University, Chongqing, China; 13 Department of Biostatistics, Southern Medical University School of Public Health, Guangzhou, China

## Abstract

**Importance:**

Measurable residual disease (MRD) is widely used as a therapy-stratification factor for acute myeloid leukemia (AML), but the association of dynamic MRD with postremission treatment (PRT) in patients with intermediate-risk AML (IR-AML) has not been well investigated.

**Objective:**

To investigate PRT choices based on dynamic MRD in patients with IR-AML.

**Design, Setting, and Participants:**

This cohort study examined 549 younger patients with de novo IR-AML in the South China Hematology Alliance database during the period from January 1, 2012, to June 30, 2016, including 154 who received chemotherapy, 116 who received an autologous stem cell transplant (auto-SCT), and 279 who received an allogeneic SCT (allo-SCT). Subgroup analyses were performed according to dynamic MRD after the first, second, and third courses of chemotherapy. The end point of the last follow-up was August 31, 2020. Statistical analysis was performed from December 1, 2019, to September 30, 2020.

**Exposures:**

Receipt of chemotherapy, auto-SCT, or allo-SCT.

**Main Outcomes and Measures:**

The primary end points were 5-year cumulative incidence of relapse and leukemia-free survival.

**Results:**

Subgroup analyses were performed for 549 participants (314 male participants [57.2%]; median age, 37 years [range, 14-60 years]) according to the dynamics of MRD after 1, 2, or 3 courses of chemotherapy. Comparable cumulative incidences of relapse, leukemia-free survival, and overall survival were observed among participants who had no MRD after 1, 2, or 3 courses of chemotherapy. Participants who underwent chemotherapy and those who underwent auto-SCT had better graft-vs-host disease–free, relapse-free survival (GRFS) than those who underwent allo-SCT (chemotherapy: hazard ratio [HR], 0.35 [95% CI, 0.14-0.90]; *P* = .03; auto-SCT: HR, 0.07 [95% CI, 0.01-0.58]; *P* = .01). Among participants with MRD after 1 course of chemotherapy but no MRD after 2 or 3 courses, those who underwent auto-SCT and allo-SCT showed lower cumulative incidence of relapse (auto-SCT: HR, 0.25 [95% CI, 0.08-0.78]; *P* = .01; allo-SCT: HR, 0.08 [95% CI, 0.02-0.24]; *P* < .001), better leukemia-free survival (auto-SCT: HR, 0.26 [95% CI, 0.10-0.64]; *P* = .004; allo-SCT: HR, 0.21 [95% CI, 0.09-0.46]; *P* < .001), and overall survival (auto-SCT: HR, 0.22 [95% CI, 0.08-0.64]; *P* = .005; allo-SCT: HR, 0.25 [95% CI, 0.11-0.59]; *P* = .001) vs chemotherapy. In addition, auto-SCT showed better GRFS than allo-SCT (HR, 0.45 [95% CI, 0.21-0.98]; *P* = .04) in this group. Among participants with MRD after 1 or 2 courses of chemotherapy but no MRD after 3 courses, allo-SCT had superior cumulative incidence of relapse (HR, 0.10 [95% CI, 0.06-0.94]; *P* = .04) and leukemia-free survival (HR, 0.18 [95% CI, 0.05-0.68]; *P* = .01) compared with chemotherapy, but no advantageous cumulative incidence of relapse (HR, 0.15 [95% CI, 0.02-1.42]; *P* = .10) and leukemia-free survival (HR, 0.23 [95% CI, 0.05-1.08]; *P* = .06) compared with auto-SCT. Among participants with MRD after 3 courses of chemotherapy, allo-SCT had superior cumulative incidences of relapse, leukemia-free survival, and overall survival compared with chemotherapy (relapse: HR, 0.16 [95% CI, 0.08-0.33]; *P* < .001; leukemia-free survival: HR, 0.19 [95% CI, 0.10-0.35]; *P* < .001; overall survival: HR, 0.29 [95% CI, 0.15-0.55]; *P* < .001) and auto-SCT (relapse: HR, 0.25 [95% CI, 0.12-0.53]; *P* < .001; leukemia-free survival: HR, 0.35 [95% CI, 0.18-0.73]; *P* = .004; overall survival: HR, 0.54 [95% CI, 0.26-0.94]; *P* = .04). Among participants with recurrent MRD, allo-SCT was also associated with advantageous cumulative incidence of relapse, leukemia-free survival, and overall survival compared with chemotherapy (relapse: HR, 0.12 [95% CI, 0.04-0.33]; *P* < .001; leukemia-free survival: HR, 0.24 [95% CI, 0.10-0.56]; *P* = .001; overall survival: HR, 0.31 [95% CI, 0.13-0.75]; *P* = .01) and auto-SCT (relapse: HR, 0.28 [95% CI, 0.09-0.81]; *P* = .02; leukemia-free survival: HR, 0.30 [95% CI, 0.12-0.76]; *P* = .01; overall survival: HR, 0.26 [95% CI, 0.10-0.70]; *P* = .007).

**Conclusions and Relevance:**

This study suggests that clinical decisions based on dynamic MRD might be associated with improved therapy stratification and optimized PRT for patients with IR-AML. Prospective multicenter trials are needed to further validate these findings.

## Key Points

**Question:**

Is there an association between dynamic measurable residual disease, treatment, and outcomes among adults with intermediate-risk acute myeloid leukemia?

**Findings:**

In this registry-based cohort study that included 549 younger patients (aged 14-60 years) with intermediate-risk acute myeloid leukemia, 154 received chemotherapy, 116 received an autologous stem cell transplant, and 279 received an allogeneic stem cell transplant. Results showed that making a postremission treatment choice based on dynamic measurable residual disease was associated with improved outcomes in subgroup analyses.

**Meaning:**

This study suggests that clinical decisions based on dynamic measurable residual disease might be associated with improved therapy stratification and optimized postremission treatment for patients with intermediate-risk acute myeloid leukemia.

## Teaser

This registry-based cohort study investigates postremission treatment choices and outcomes based on dynamic measurable residual disease in adults with intermediate-risk acute myeloid leukemia (AML).

## Introduction

Most younger patients with acute myeloid leukemia (AML) can achieve complete remission (CR) with induction chemotherapy.^1^ After CR, further postremission treatment (PRT) would be necessary to prevent relapse.^2^ Postremission treatment usually consists of either a stem cell transplant (SCT) or cytarabine-based consolidation chemotherapy. Allogeneic SCT (allo-SCT) is generally accepted as the most effective PRT to prevent relapse, but it has relatively high rates of transplant-related mortality.^3^ Autologous SCT (auto-SCT) and chemotherapy carry a lower risk of transplant-related mortality compared with allo-SCT.^4^ However, this lower risk of transplant-related mortality is offset by a higher risk of relapse owing to the lack of a graft-vs-leukemia effect and the potential infusion of leukemia cells in grafts.^5,6^ Currently, decisions for PRT depend mainly on risk stratification based on cytogenetics and molecular markers,^2,7^ but it remains controversial, particularly for patients with intermediate-risk AML (IR-AML).^3,4,5,6,8,9,10,11,12,13^ For instance, some studies have suggested that a survival benefit is associated with the use of allo-SCT in patients with IR-AML,^3,5,6^ whereas other studies have showed that auto-SCT or chemotherapy had no significant survival differences compared with allo-SCT.^4,12,13^ Thus, these studies highlight the need to find additional factors associated with survival outcomes that optimize PRT choices for patients with IR-AML.

An increasing number of studies have demonstrated that measurable residual disease (MRD) is independently associated with relapse and with survival among patients with AML.^14,15,16,17,18,19,20^ Several studies have suggested that risk stratification by the combination of genetics and MRD might optimize PRT.^19,20,21^ However, these studies focused mainly on MRD at a specific time point. It might be best to think of MRD as a dynamic variable during the process of therapeutic decision-making.^22,23,24^ In this study, we retrospectively analyzed a large data set to explore the clinical significance of dynamic MRD on directing PRT for younger patients with IR-AML in first CR (CR1).

## Methods

### Study Population

This registry-based cohort study examined all consecutive patients with de novo AML in the South China Hematology Alliance database between January 1, 2012, and June 30, 2016. Patients were enrolled if they (1) had received a diagnosis of IR-AML, (2) were in CR1, and (3) were 14 to 60 years of age. Exclusion criteria included the following: (1) acute promyelocytic leukemia, (2) *NPM1* variant with an FLT3 internal tandem duplication, (3) failure to to achieve CR after 2 cycles of induction chemotherapy, (4) less than 3 cycles of consolidation in the chemotherapy group, and (5) lack of MRD parameters. The diagnosis of IR-AML was based on National Comprehensive Cancer Network criteria.^2^ Patients with an FLT3 internal tandem duplication variant were excluded because they received sorafenib tosylate treatment. According to the PRT strategy, patients were classified into 3 groups: chemotherapy, auto-SCT, and allo-SCT. Patients were followed up via telephone or outpatient visit by treating physicians. The end point of the last follow-up was August 31, 2020. This study was approved by the ethical review boards of Nanfang Hospital, Sun Yat-Sen Memorial Hospital, Guangdong Second Provincial General Hospital, Guangzhou First People’s Hospital, The First People’s Hospital of Chenzhou, The First Affiliated Hospital of Zhengzhou University, Shenzhen Hospital of Southern Medical University, Shenzhen Hospital of Peking University, The First Affiliated Hospital of Guangxi Medical University, Zhongshan People’s Hospital, and The First Affiliated Hospital of Chongqing Medical University. Written informed consent was obtained from participants in accordance with the modified Helsinki Declaration.^25^ This study followed the Strengthening the Reporting of Observational Studies in Epidemiology (STROBE) reporting guideline.^26^

### Treatment Procedures

According to our practical guidelines, patients are generally scheduled for induction therapy consisting of daunorubicin hydrochloride, 60 mg/m^2^, or idarubicin hydrochloride, 10 to 12 mg/m^2^, on days 1 to 3 and cytarabine, 200 mg/m^2^ per day, for 7 days. For patients who failed to achieve CR after the first induction regimen, a second induction regimen consisting of daunorubicin hydrochloride, 60 mg/m^2^, or idarubicin hydrochloride, 10 mg/m^2^ per day, on days 1 to 3 and cytarabine, 2.0 g/m^2^ twice daily, on days 1 to 3, or the same regimen as the first induction regimen was administered. After CR, usually 4 courses of cytarabine-based consolidation chemotherapy, 3 courses of chemotherapy followed by auto-SCT, or 2 courses of chemotherapy followed by allo-SCT were administered based on MRD status and donor availability. In auto-SCT, peripheral blood stem cell grafts were collected after mobilization with etoposide plus intermediate-dose cytarabine combined with granulocyte colony-stimulating factor. In allo-SCT, the principle of donor selection was based on the consensus in China.^27^ Busulfan-based myeloablative conditioning regimens were used for all patients.^28^ The prophylaxis for graft-vs-host disease (GVHD) was described previously.^29^

### Cytogenetic and Molecular Assessment

Cytogenetic analyses were performed with Giemsa staining and reverse banding techniques and fluorescence in situ hybridization. Molecular markers were detected by polymerase chain reaction and next-generation sequencing.

### MRD Detection

Generally, MRD in bone marrow was assessed after induction therapy and each cycle of PRT and then at 2-month intervals within the first year, 3-month intervals within the second year, 4-month intervals within the third year, and half-year intervals from the fourth to fifth year after PRT. Multiparameter flow cytometry was routinely used for MRD monitoring.^30,31^ The MRD level of 0.1% was used as a threshold to distinguish MRD positive from MRD negative.

### Evaluation End Points and Definition

Evaluation end points included relapse, survival, and transplant-related mortality. Complete remission was defined as less than 5% blasts by morphologic evaluation of the bone marrow with no evidence of dysplasia in the bone marrow and no manifestation of leukemia outside the hematopoietic system. Relapse was defined by morphologic evidence in the peripheral blood, bone marrow, or extramedullary sites. Transplant-related mortality was estimated as death without evidence of leukemia recurrence. Leukemia-free survival was calculated from CR1 to relapse or death. Overall survival (OS) was calculated from CR1 to death. Graft-vs-host-disease–free, relapse-free survival (GRFS) events were defined as grade III or IV acute GVHD, chronic GVHD requiring systemic immunosuppressive treatment, leukemia relapse, or death from any cause from CR1 to last follow-up; GRFS in the chemotherapy and auto-SCT groups was considered equal to leukemia-free survival because of no incidence of GVHD in both groups, and it was compared with that in allo-SCT. Patients who did not achieve morphologic CR after the first induction regimen were considered to have MRD.

### Statistical Analysis

Statistical analysis was performed from December 1, 2019, to September 30, 2020. Variables associated with patients, disease, and transplant characteristics among groups were compared using the Fisher exact test for categorical variables and the Mann-Whitney test for continuous variables. Numerical variables were analyzed as categories based on their values being below or above the median of the entire cohort. Overall survival, leukemia-free survival, and GRFS were estimated using the Kaplan-Meier method and compared by the log-rank test. Cumulative incidence curves were used in a competing risk setting with relapse treated as a competing event to calculate transplant-related mortality probabilities and with transplant-related mortality treated as a competing risk to calculate relapse. The correlations between MRD at different time points was analyzed by the Spearman test. The Cox proportional hazards regression model was used for the analysis of risk factors for time-to-event variables. The Fine-Gray model was used for analysis of end points involving competing risks.^32^ All tests were 2-sided, with significance set at *P* = .05. Stata SE, version 12.0 (StataCorp LP) and R, version 3.4.3 (R Group for Statistical Computing) were used for all data analysis.

## Results

### Patient Demographic Characteristics and Treatment Characteristics

**Figure 1.**
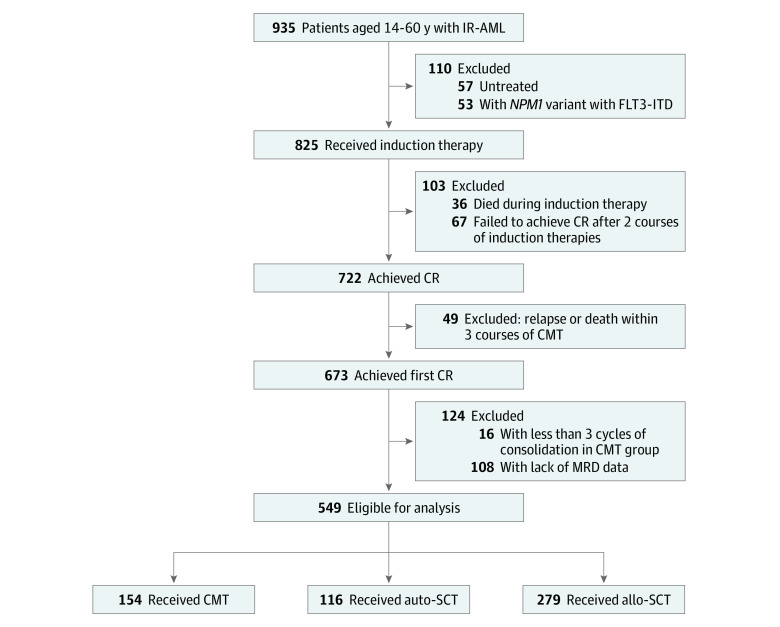
Flow Diagram of Study Participants Allo-SCT indicates allogeneic stem cell transplant; auto-SCT, autologous stem cell transplant; CMT, chemotherapy; CR, complete remission; IR-AML, intermediate-risk acute myeloid leukemia; ITD, internal tandem duplication; and MRD, measurable residual disease.

A total of 549 consecutive patients (314 male patients [57.2%] and 235 female patients [42.8%]; median age, 37 years [range, 14-60 years]) were enrolled in this study, including 154 in the chemotherapy group, 116 in the auto-SCT group, and 279 in the allo-SCT group (Figure 1). In the allo-SCT group, 133 patients received transplants from matched sibling donors (MSDs), and 146 patients received transplants from alternative donors (ADs) (including 97 haploidentical donors and 45 suitably matched unrelated donors; 4 patients received umbilical cord blood). The median age of patients in the chemotherapy group was 47 years (range, 14-60 years), the median age of patients in the auto-SCT group was 33.5 years (range, 15-60 years), and the median age of patients in the allo-SCT group was 35 years (range, 14-60 years). Patient demographic and treatment characteristics for the 3 groups are presented in Table 1.

**Table 1.** Demographic and Treatment Characteristics of Patients

Characteristic	Patients, No. (%)			*P* value
	CMT (n = 154)	Auto-SCT (n = 116)	Allo-SCT (n = 279)	
Age, median (range), y	47 (14-60)	33.5 (15-60)	35 (14-60)	<.001
Sex				
Male	85 (55.2)	74 (63.8)	155 (55.6)	.27
Female	69 (44.8)	42 (36.2)	124 (44.4)	
WBC count, median (range), /µL	19 400 (800-407 000)	21 400 (1900-325 000)	20 300 (600-376 300)	.73
First induction therapy				
DA	42 (27.3)	29 (25.0)	60 (21.5)	.34
IA	89 (57.8)	70 (60.3)	188 (67.4)	
Other	23 (14.9)	17 (14.7)	31 (11.1)	
Second induction therapy or first consolidation therapy				
DA	14 (9.1)	8 (6.9)	16 (5.7)	.81
IA	14 (9.1)	13 (11.2)	21 (7.5)	
3 + 3	48 (31.2)	31 (26.7)	80 (28.7)	
Intermediate- or high-dose cytarabine	68 (44.2)	56 (48.3)	144 (51.6)	
Others	10 (6.5)	8 (6.9)	18 (6.5)	
No. of cycles of consolidation chemotherapy, median (range)	4 (3-7)	3 (3-5)	2 (1-4)	NA
Duration from CR1 to transplant, median (range), mo	NA	5.9 (5.4-9.6)	3.9 (2.8-7.7)	<.001
No. of cycles to CR				
1	126 (81.8)	97 (83.6)	217 (77.8)	.34
2	28 (18.2)	19 (16.4)	62 (22.2)	
MRD1				
Positive	81 (52.6)	64 (55.2)	215 (77.1)	<.001
Negative	73 (47.4)	52 (44.8)	64 (22.9)	
MRD2				
Positive	52 (33.8)	33 (28.4)	132 (47.3)	.001
Negative	102 (66.2)	83 (71.6)	147 (52.7)	
MRD3				
Positive	39 (25.3)	26 (22.4)	105 (37.6)	.002
Negative	115 (74.7)	90 (77.6)	174 (62.4)	

Abbreviations: 3 + 3, daunorubicin hydrochloride or idarubicin hydrochloride, 10 mg/m^2^ per day, on days 1 to 3 and cytarabine, 2.0 g/m^2^ twice daily, on days 1 to 3; allo-SCT, allogeneic stem cell transplant; auto-SCT, autologous stem cell transplant; CMT, chemotherapy; CR, complete remission; CR1, first CR; DA, daunorubicin hydrochloride and cytarabine; IA, idarubicin hydrochloride and cytarabine; MRD1, measurable residual disease after 1 course of CMT; MRD2, measurable residual disease after 2 courses of CMT; MRD3, measurable residual disease after 3 courses of CMT; NA, not applicable.

SI conversion factor: To convert WBC to ×10^9^ per liter, multiply by 0.001.

### Relapse and Survival

**Figure 2.**
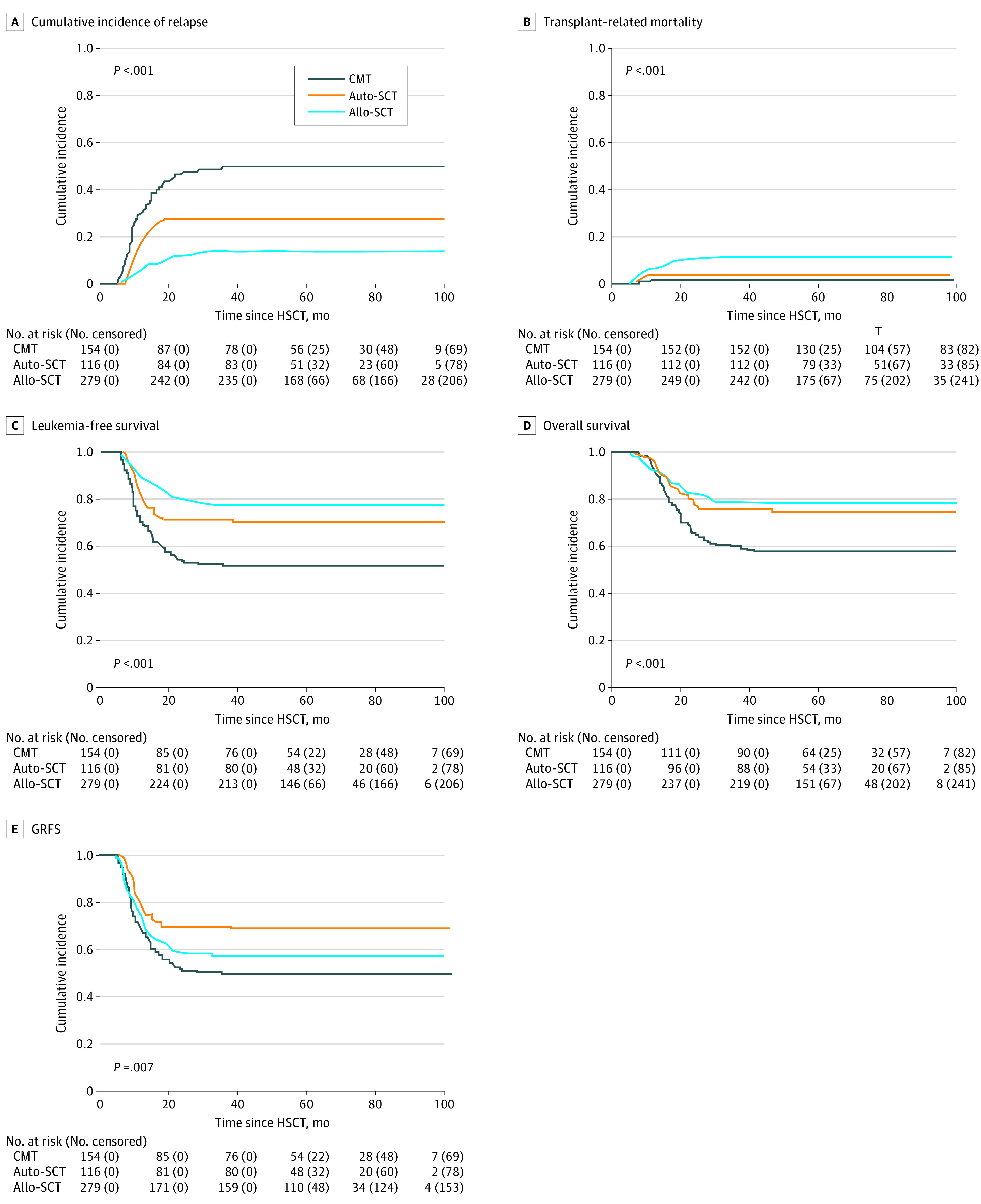
Outcomes for All Patients Based on Postremission Treatment Allo-SCT indicates allogeneic stem cell transplant; auto-SCT, autologous stem cell transplant; CMT, chemotherapy; GRFS, graft-vs-host disease–free, relapse-free surviva; HSCT, hematopoietic stem cell transplant; and SCT, stem cell transplant.

At last follow-up, 146 patients had relapsed. The median time from CR1 to relapse of the total cohort was 10.9 months (range, 5.4-42.4 months), the median time from CR1 to relapse of patients in the chemotherapy group was 9.9 months (range, 5.4-35.4 months), the median time from CR1 to relapse of patients in the auto-SCT group was 10.9 months (range, 7.2-38.2 months), and the median time from CR1 to relapse of patients in the allo-SCT group was 13.0 months (range, 7.4-42.4 months) (*P* = .03). The time from CR1 to relapse was much longer in the allo-SCT group compared with the chemotherapy group (*P* = .01), but no significant difference between the allo-SCT and auto-SCT groups (*P* = .09) or between the auto-SCT and chemotherapy groups (*P* = .38) was found. Of the 76 patients in the chemotherapy group who relapsed, 58 received further treatment (23 allo-SCT), and 13 survived at the last follow-up. Of the 32 patients in the auto-SCT group who relapsed, 26 received further treatment (15 allo-SCT), and 7 survived at the last follow-up. Of the 38 patients in the allo-SCT group who relapsed, 29 received salvage treatment, and 6 survived at the last follow-up. The 5-year cumulative incidence of relapse was 49.4% (95% CI, 41.2%-57.0%) in the chemotherapy group, 27.6% (95% CI, 19.8%-36.0%) in the auto-SCT group, and 13.6% (95% CI, 9.9%-17.9%) in the allo-SCT group (*P* < .001) (Figure 2A). Patients in the allo-SCT group showed significantly lower cumulative incidence of relapse than those in the auto-SCT group (hazard ratio [HR], 0.44 [95% CI, 0.28-0.71]; *P* = .001) and chemotherapy group (HR, 0.21 [95% CI, 0.14-0.31]; *P* < .001), and those in the auto-SCT group had lower cumulative incidence of relapse than those in the chemotherapy group (HR, 0.47 [95% CI, 0.31-0.71]; *P* < .001). For patients who underwent allo-SCT, the cumulative incidence of relapse was not significantly different between patients who received transplants from ADs (AD-SCT) and those who received transplants from MSDs (MSD-SCT) (HR, 0.59 [95% CI, 0.31-1.12]; *P* = .11).

With a median follow-up of 48.1 months (range, 5.4-94.7 months) after CR1, 394 patients survived and 155 died. The causes of death are shown in the eTable in the Supplement. The 5-year cumulative incidence of transplant-related mortality was 1.3% (95% CI, 0.3%-4.2%) in the chemotherapy group, 3.4% (95% CI, 1.1%-8.0%) in the auto-SCT group, and 10.4% (95% CI, 7.2%-14.3%) in the allo-SCT group (*P* < .001) (Figure 2B). Patients in the allo-SCT group had a higher rate of transplant-related mortality than those in the chemotherapy group (HR, 9.36 [95% CI, 1.99-35.07]; *P* = .004) and those in the auto-SCT group (HR, 3.10 [95% CI, 1.08-8.86]; *P* = .04), whereas there was no significant difference between patients in the auto-SCT and chemotherapy groups (HR, 2.70 [95% CI, 0.49-14.78]; *P* = .25). The 5-year leukemia-free survival rate was 49.3% (95% CI, 41.2%-57.0%) in the chemotherapy group, 69.0% (95% CI, 59.7%-76.5%) in the auto-SCT group, and 76.0% (95% CI, 70.5%-80.6%) in the allo-SCT group (*P* < .001) (Figure 2C). Leukemia-free survival was significantly higher among patients in the auto-SCT and allo-SCT groups compared with those in the chemotherapy (auto-SCT: HR, 0.53 [95% CI, 0.36-0.78]; *P* = .002; and allo-SCT: HR, 0.38 [95% CI, 0.27-0.53]; *P* < .001), but it was comparable between the allo-SCT and auto-SCT groups (HR, 0.72 [95% CI, 0.48-1.08]; *P* = .11). The 5-year OS rate was 57.6% (95% CI, 49.4%-65.0%) in the chemotherapy group, 75.0% (95% CI, 66.1%-81.9%) in the auto-SCT group, and 78.1% (95% CI, 72.8%-82.5%) in the allo-SCT group (*P* < .001) (Figure 2D). Overall survival was significantly better in the auto-SCT and allo-SCT groups than in the chemotherapy group (auto-SCT: HR, 0.54 [95% CI, 0.35-0.84]; *P* = .006; allo-SCT: HR, 0.47 [95% CI, 0.33-0.66]; *P* < .001, respectively), but it was comparable between the allo-SCT and auto-SCT groups (HR, 0.87 [95% CI, 0.56-1.35]; *P* = .52). The 5-year GRFS rate was 49.3% (95% CI, 41.2%-57.0%) in the chemotherapy group, 69.0% (95% CI, 59.7%-76.5%) in the auto-SCT group, and 56.5% (95% CI, 50.5%-62.1%) in the allo-SCT group (*P* = .007) (Figure 2E). Patients in the auto-SCT group had better GRFS than those in the chemotherapy group (HR, 0.54 [95% CI, 0.36-0.79]; *P* = .002) and those in the allo-SCT group (HR, 0.65 [95% CI, 0.45-0.94]; *P* = .02), but there was no significant difference between patients in the allo-SCT group and patients in the chemotherapy group (HR, 0.82 [95% CI, 0.62-1.09]; *P* = .17). In addition, subgroup analysis showed that, compared with patients who received MSD-SCT, those who received AD-SCT did not have higher transplant-related mortality (HR, 2.11 [95% CI, 0.97-4.61]; *P* = .06) but did have similar leukemia-free survival (HR, 1.04 [95% CI, 0.64-1.67]; *P* = .89), OS (HR, 1.06 [95% CI, 0.64-1.75]; *P* = .83), and GRFS rates (HR, 1.35 [95% CI, 0.94-1.94]; *P* = .10).

### Multivariate Analysis of Relapse and Survival

The univariate and multivariate analyses of relapse, leukemia-free survival, and OS are presented in Table 2. Competing risk regression showed that needing 2 cycles to achieve CR and having MRD after 3 courses of chemotherapy were risk factors for relapse in the entire population. Cox proportional hazards regression analysis revealed that needing 2 cycles to achieve CR and having MRD after 3 cycles of chemotherapy were also risk factors for leukemia-free survival and OS. Allo-SCT had a beneficial association with relapse, leukemia-free survival, and OS in multivariate analysis when taking auto-SCT or chemotherapy as a reference. In addition, auto-SCT had a beneficial association with relapse, leukemia-free survival, and OS when taking chemotherapy as a reference.

**Table 2.** Multivariate Analysis for Relapse, Leukemia-Free Survival, and Overall Survival

Variable	Relapse		Leukemia-free survival		Overall survival	
	HR (95% CI)	*P* value	HR (95% CI)	*P* value	HR (95% CI)	*P* value
Age, ≥37 y vs <37 y	1.02 (0.71-1.45)	.89	1.02 (0.71-1.47)	.93	1.19 (0.84-1.68)	.33
Sex, female vs male	1.04 (0.73-1.46)	.85	1.12 (0.79-1.58)	.65	1.09 (0.79-1.51)	.59
WBC count, ≥20 000/µL vs <20 000/µL	1.33 (0.93-1.90)	.12	1.27 (1.00-1.79)	.18	1.20 (0.86-1.66)	.29
Cycles to achieve CR, 2 vs 1	1.81 (1.19-2.76)	.006	1.97 (1.33-2.90)	.001	1.78 (1.23-2.58)	.002
MRD1	1.24 (0.44-3.56)	.68	1.11 (0.42-3.04)	.81	1.35 (0.48-3.78)	.57
MRD2	1.47 (0.92-2.36)	.19	1.49 (0.95-2.33)	.19	1.32 (0.86-2.02)	.28
MRD3	3.93 (1.55-9.97)	.004	3.93 (1.65-9.37)	.002	6.21 (2.47-15.63)	<.001
PRT						
Auto-SCT vs CMT	0.49 (0.31-0.78)	.002	0.50 (0.32-0.79)	.003	0.60 (0.38-0.94)	.03
Allo-SCT vs CMT	0.12 (0.08-0.19)	<.001	0.12 (0.08-0.19)	<.001	0.33 (0.23-0.48)	<.001
Allo-SCT vs auto-SCT	0.25 (0.16-0.41)	<.001	0.24 (0.15-0.39)	<.001	0.56 (0.36-0.88)	.01

Abbreviations: allo-SCT, allogeneic stem cell transplant; auto-SCT, autologous stem cell transplant; CMT, chemotherapy; CR, complete remission; HR, hazard ratio; MRD1, measurable residual disease after 1 course of CMT; MRD2, measurable residual disease after 2 courses of CMT; MRD3, measurable residual disease after 3 courses of CMT; PRT, postremission treatment; WBC, white blood cell.

### Dynamic MRD and Direct PRT Selection

To explore the association of dynamic MRD with PRT selection for patients with IR-AML, subgroup analyses were performed according to the dynamics of MRD after 1 course of chemotherapy, MRD after 2 courses of chemotherapy, and MRD after 3 courses of chemotherapy. Patients were classified into the following 5 subgroups: (1) subgroup A, defined as being persistent MRD negative after the first induction regimen for all 3 courses; (2) subgroup B, defined as being persistent MRD positive after all 3 courses; (3) subgroup C, defined as being recurrent MRD positive after being MRD negative; (4) subgroup D, defined as being MRD negative after 2 courses of chemotherapy; and (5) subgroup E, defined as being MRD negative after 3 courses of chemotherapy. The univariate analysis of each subgroup is presented in Table 3. Furthermore, subgroup analyses were performed after adjustment for various covariates, including age, sex, white blood cell count, and number of cycles to achieve CR.

**Table 3.** Outcomes of Patients (at 5 Years) Receiving Different PRT Based on Dynamics of MRD1, MRD2, and MRD3

MRD status and PRT	No.	% (95% CI)					
		Overall survival	Leukemia-free survival	CIR	TRM	GRFS
Subgroup A^a^						
CMT	55	93.6 (81.5-98.0)	87.3 (75.2-93.7)	12.7 (5.5-23.0)	0	87.3 (75.2-93.7)
Auto-SCT	37	97.3 (82.3-99.6)	97.3 (82.3-99.6)	2.7 (0.2-12.3)	0	97.3 (82.3-99.6)
Allo-SCT	41	88.5 (68.4-96.1)	90.3 (76.1-96.2)	2.4 (0.2-11.2)	7.3 (1.9-18.0)	70.7 (54.3-82.2)
*P* value		.40	.27	.07	.03^b^	.003
Subgroup B						
CMT	21	14.3 (3.6-32.1)	4.8 (0.3-19.7)	90.5 (59.8-98.1)	4.7 (0.3-20.5)	4.8 (0.3-19.7)
Auto-SCT	16	37.5 (15.4-59.8)	25.0 (7.8-47.2)	68.8 (38.0-86.5)	6.3 (0.4-25.7)	25.0 (7.8-47.2)
Allo-SCT	77	64.9 (53.2-74.4)	61.0 (49.2-70.9)	26.0 (16.7-36.2)	13.0 (6.6-21.6)	44.2 (32.9-54.8)
*P* value		<.001	<.001	<.001	.48	<.001
Subgroup C						
CMT	22	27.3 (11.1-46.4)	18.2 (5.7-36.3)	81.8 (56.4-93.2)	0	18.2 (5.7-36.3)
Auto-SCT	17	41.2 (18.6-62.6)	35.3 (14.5-57.0)	52.9 (26.2-73.9)	11.8 (1.8-32.0)	35.3 (14.5-57.0)
Allo-SCT	31	71.0 (51.6-83.7)	67.7 (48.4-81.2)	22.6 (9.8-38.6)	9.7 (2.4-23.2)	48.4 (30.2-64.4)
*P* value		.02	.001	<.001	.28	.10
Subgroup D						
CMT	35	51.4 (34.0-66.4)	45.7 (28.9-61.1)	51.4 (33.6-66.7)	2.9 (0.2-13.0)	45.7 (28.9-61.1)
Auto-SCT	38	86.8 (71.2-94.3)	78.9 (62.3-88.9)	21.1 (9.8-35.2)	0	78.9 (62.3-88.9)
Allo-SCT	85	87.1 (77.9-92.6)	84.7 (75.1-90.8)	5.9 (2.2-12.3)	9.4 (4.4-16.8)	64.5 (53.3-73.7)
*P* value		<.001	<.001	<.001	.08^c^	.03
Subgroup E						
CMT	21	45.8 (23.4-65.8)	33.3 (14.9-53.1)	66.7 (41.2-83.1)	0	33.3 (14.9-53.1)
Auto-SCT	8	62.5 (22.9-86.1)	50.0 (15.2-77.5)	37.5 (7.0-69.7)	12.5 (0.4-45.3)	50.0 (15.2-77.5)
Allo-SCT	45	77.8 (62.6-87.4)	77.8 (62.6-87.4)	11.1 (4.0-22.3)	11.1 (4.0-22.3)	55.6 (40.0-68.6)
*P* value		.02	<.001	<.001	.29	.14

Abbreviations: allo-SCT, allogeneic stem cell transplant; auto-SCT, autologous stem cell transplant; CIR, cumulative incidence of relapse; CMT, chemotherapy; GRFS, graft-vs-host disease–free, relapse-free survival; MRD, measurable residual disease; MRD1, measurable residual disease after 1 course of CMT; MRD2, measurable residual disease after 2 courses of CMT; MRD3, measurable residual disease after 3 courses of CMT; PRT, postremission treatment; TRM, transplant-related mortality.

^a^ Subgroup A, patients who became MRD negative after 1 course of CMT and were persistently MRD negative; subgroup B, persistently positive MRD; subgroup C, recurrent MRD-positive patients after being MRD negative; subgroup D, patients who become MRD negative after 2 courses of CMT; and subgroup E, patients who become MRD negative after 3 courses of CMT.

^b^ CMT vs allo-SCT (*P* = .04) and auto-SCT vs allo-SCT (*P* = .09).

^c^ Auto-SCT vs allo-SCT (*P* = .04).

In subgroup A, comparable cumulative incidence of relapse (HR, 0.37 [95% CI, 0.11-1.20]; *P* = .10), leukemia-free survival (HR, 0.83 [95% CI, 0.41-1.66]; *P* = .59), and OS (HR, 1.48 [95% CI, 0.64-3.45]; *P* = .36) were found among the 3 different PRT groups. In addition, better GRFS was observed for those in the chemotherapy group (HR, 0.35 [95% CI, 0.14-1.00]; *P* = .03) and the auto-SCT group (HR, 0.07 [95% CI, 0.01-0.58]; *P* = .01) compared with the allo-SCT group, whereas GRFS was comparable between those in the auto-SCT and chemotherapy groups (HR, 0.21 [95% CI, 0.03-1.74]; *P* = .15).

In subgroup B, patients in the allo-SCT group had a lower cumulative incidence of relapse than those in the chemotherapy group (HR, 0.16 [95% CI, 0.08-0.33]; *P* < .001) and those in the auto-SCT group (HR, 0.25 [95% CI, 0.12-0.53]; *P* < .001), as well as better leukemia-free survival and OS compared with the chemotherapy group (leukemia-free survival: HR, 0.19 [95% CI, 0.10-0.35]; *P* < .001; OS: HR, 0.30 [95% CI, 0.15-0.55]; *P* < .001) and the auto-SCT group (leukemia-free survival: HR, 0.35 [95% CI, 0.19-0.73]; *P* = .004; OS: HR, 0.54 [95% CI, 0.26-0.94]; *P* = .04).

In subgroup C, patients in the allo-SCT group had better leukemia-free survival and OS compared with patients in the chemotherapy group (leukemia-free survival: HR, 0.24 [95% CI, 0.10-0.56]; *P* = .001; OS: HR, 0.31 [95% CI, 0.13-0.75]; *P* = .01) and patients in the auto-SCT group (leukemia-free survival: HR, 0.30 [95% CI, 0.12-0.76]; *P* = .01; OS: HR, 0.26 [95% CI, 0.10-0.70]; *P* = .007) owing to a lower cumulative incidence of relapse (chemotherapy: HR, 0.12 [95% CI, 0.04-0.33]; *P* < .001; auto-SCT: HR, 0.28 [95% CI, 0.09-0.81]; *P* = .02).

In subgroup D, patients who underwent auto-SCT had a significantly lower cumulative incidence of relapse and better leukemia-free survival and OS compared with those in the chemotherapy group (cumulative incidence of relapse: HR, 0.25 [95% CI, 0.08-0.78]; *P* = .01; leukemia-free survival: HR, 0.26 [95% CI, 0.10-0.64]; *P* = .004; OS: HR, 0.22 [95% CI, 0.08-0.64]; *P* = .005) and those in the allo-SCT group (cumulative incidence of relapse: HR, 0.08 [95% CI, 0.02-0.24]; *P* < .001; leukemia-free survival: HR, 0.21 [95% CI, 0.09-0.46]; *P* < .001; OS: HR, 0.25 [95% CI, 0.11-0.59]; *P* = .001). Patients in the allo-SCT group had a lower cumulative incidence of relapse than those in the auto-SCT group (HR, 0.31 [95% CI, 0.09-0.96]; *P* = .04) but comparable leukemia-free survival (HR, 1.24 [95% CI, 0.51-3.00]; *P* = .64) and OS (HR, 0.86 [95% CI, 0.30-2.49]; *P* = .78). In addition, patients in the auto-SCT group achieved better GRFS compared with those in the allo-SCT group (HR, 0.45 [95% CI, 0.21-0.98]; *P* = .04).

In subgroup E, patients in the allo-SCT group had a lower cumulative incidence of relapse and better leukemia-free survival compared with those in the chemotherapy group (cumulative incidence of relapse: HR, 0.10 [95% CI, 0.06-0.94]; *P* = .04; leukemia-free survival: HR, 0.18 [95% CI, 0.05-0.68]; *P* = .01), whereas OS was comparable between the 2 groups (HR, 0.42 [95% CI, 0.14-1.31]; *P* = .13). Patients in the allo-SCT group did not have a lower cumulative incidence of relapse (HR, 0.15 [95% CI, 0.02-1.42]; *P* = .10), better leukemia-free survival (HR, 0.23 [95% CI, 0.05-1.08]; *P* = .06), or difference in OS (HR, 0.46 [95% CI, 0.10-2.18]; *P* = .33) compared with those in the auto-SCT group. In addition, exploratory subgroup analyses of survival revealed similar leukemia-free survival, OS, and GRFS between patients who received AD-SCT and those who received MSD-SCT in all 5 subgroups (eFigure in the Supplement).

## Discussion

This study aimed to explore PRT choices based on dynamic MRD for younger patients with IR-AML. The results suggest the following: (1) chemotherapy and auto-SCT were associated with better GRFS vs allo-SCT for patients who were persistently MRD negative after the first induction regimen; (2) patients who were persistently MRD positive and those with recurrent MRD who received allo-SCT had better leukemia-free survival and OS than those who received auto-SCT or chemotherapy; (3) patients who were MRD negative after 2 cycles of chemotherapy had better leukemia-free survival and OS with auto-SCT and allo-SCT than with chemotherapy, whereas those receiving auto-SCT showed better GRFS compared with those receiving allo-SCT; and (4) for patients who were MRD negative after 3 cycles of chemotherapy, allo-SCT had comparatively more favorable survival outcomes. To our knowledge, this is the first attempt to investigate PRT strategies based on dynamic MRD for patients with IR-AML.

It remains a challenge for practitioners to choose the optimal PRT for patients with IR-AML because different conclusions have been drawn in previous reports.^3,4,8,9,10,11,12,13,33^ On the one hand, some studies have suggested that patients with IR-AML might benefit from allo-SCT. For instance, MSD-SCT or matched unrelated donor–SCT might be superior to chemotherapy or auto-SCT.^3,6,11,33^ In addition, some investigators have reported that haploidentical donor–SCT also achieved more favorable survival outcomes than chemotherapy or auto-SCT owing to significantly lower cumulative incidence of relapse and acceptable transplant-related mortality.^5,34,35^ On the other hand, some studies have indicated that chemotherapy or auto-SCT might achieve comparable survival compared with allo-SCT because the disadvantage in a higher cumulative incidence of relapse in the former was offset by lower transplant-related mortality.^9,10,12,13^ The controversies in this issue highlight the limited therapy-directing ability of genetics alone and the need for integrating predictive factors, such as MRD monitoring, after diagnosis. Measurable residual disease has been extensively used as a therapy-stratification parameter for AML.^19,20,21,36,37,38^ Recently, the GIMEMA AML1310 trial developed a strategy of making PRT choices based on MRD after 1 consolidation for patients with IR-AML.^21^ In that trial, MRD-negative patients were to receive auto-SCT, while MRD-positive patients were to receive allo-SCT; similar OS and leukemia-free survival were observed between both groups of patients. However, the best timing for treatment choice based on MRD is still debatable.^23,39^ Freeman et al^20^ reported that MRD after induction therapy could improve outcome stratification by redefining partial response for IR-AML. However, Yao et al^37^ and Venditti et al^21^ suggested that MRD after 1 consolidation might be the best timing for choosing PRT. Lv et al^5^ reported that MRD after the second consolidation was independently associated with cumulative incidence of relapse, leukemia-free survival, and OS for patients with IR-AML. These studies all focused on the value of static MRD for prognosis and treatment choice.

In our study, allo-SCT and auto-SCT showed advantageous cumulative incidence of relapse, leukemia-free survival, and OS compared with chemotherapy, and allo-SCT had lower cumulative incidence of relapse but higher transplant-related mortality compared with auto-SCT, with comparable leukemia-free survival and OS in the entire population. Further subgroup analyses based on dynamic MRD showed that chemotherapy and auto-SCT had comparable cumulative incidence of relapse, leukemia-free survival, and OS as allo-SCT but better GRFS than allo-SCT owing to lower transplant-related mortality and no GVHD in patients who were persistently MRD negative. For patients who were persistently MRD positive and patients with recurrent MRD, allo-SCT had better leukemia-free survival and OS than chemotherapy, and auto-SCT had a lower cumulative incidence of relapse. For the patients who were MRD negative after 2 cycles of chemotherapy, auto-SCT and allo-SCT had superior cumulative incidence of relapse, leukemia-free survival, and OS compared with chemotherapy. Allo-SCT had a lower cumulative incidence of relapse but higher transplant-related mortality than auto-SCT and comparable leukemia-free survival and OS between the 2 groups. However, auto-SCT had better GRFS than allo-SCT. For patients who were MRD negative after 3 cycles of chemotherapy, allo-SCT had a better cumulative incidence of relapse and leukemia-free survival than chemotherapy. Based on these results, we suggest that chemotherapy and auto-SCT might be preferable for patients who are persistently MRD negative, and allo-SCT should be recommended for patients who are persistently MRD positive and patients with recurrent MRD. Auto-SCT prior to allo-SCT might be recommended for patients who are MRD negative after 2 cycles of chemotherapy. Allo-SCT might be preferable for patients who are MRD negative after 3 cycles of chemotherapy.

During the last decade, an increasing number of studies have shown that transplants using ADs achieve similar outcomes as MSD-SCT.^30,40,41,42,43,44^ In our study, the results revealed that AD-SCT achieved comparable leukemia-free survival, OS, and GRFS as MSD-SCT in all subgroup analyses, which was consistent with previous studies.^30,40,41,42^

Number of cycles to achieve CR was another factor associated with prognosis.^45,46^ Our results suggested that needing 2 cycles to achieve CR was an independent risk factor for relapse, leukemia-free survival, and OS, in accordance with previous studies.^45,46^ Old age was usually associated with poor prognosis because of unfavorable genetics, chemotherapeutic resistance, poor performance status, and frequent comorbidities.^47,48^ In our study, patients in the chemotherapy group were significantly older than those in the auto-SCT and allo-SCT groups. However, age was not associated with relapse or survival in univariate and multivariate analyses. The possible reason for our results might be that patients were excluded if they were older than 60 years, failed to achieve CR after 2 cycles of chemotherapy, or had received a diagnosis of poor-risk genetics.

### Limitations

This study has some limitations. Although the data came from a registered database, the bias of a retrospective study was inevitable. To address this issue and further validate our findings, we have conducted a prospective, multicenter trial on dynamic MRD-directed therapies for AML (ClinicalTrials.gov identifier: NCT02870777).

## Conclusions

Our results suggest that clinical decisions based on dynamic MRD might be associated with improved therapy stratification and optimized PRT for patients with IR-AML. CMT and auto-SCT might be preferable for the persistent MRD-negative patients, and allo-SCT should be strongly recommended for the persistent MRD-positive and recurrent MRD-positive patients. Auto-SCT prior to allo-SCT might be recommended for the patients who were MRD negative after 2 cycles of chemotherapy. Allo-SCT might be preferable for the patients who were MRD negative after 3 cycles of chemotherapy. Prospective multicenter trials are needed to further validate our findings.

## Notes

Supplement. **eTable.** Causes of Death Posttransplantation **eFigure.** Forest Plots for Survival of MSD-SCT and AD-SCT
