# Supplementary material for: Association Between Measurable Residual Disease in Patients With Intermediate-Risk Acute Myeloid Leukemia and First Remission, Treatment, and Outcomes
Source: JAMA Netw Open. 2021 Jul 7;4(7):e2115991. doi: 10.1001/jamanetworkopen.2021.15991 (PMC8264648; doi:10.1001/jamanetworkopen.2021.15991)
Supplement: Supplement. — eTable. Causes of Death Posttransplantation eFigure. Forest Plots for Survival of MSD-SCT and AD-SCT [file jamanetwopen-e2115991-s001.pdf]

Supplementary Online Content

Yu S, Fan Z, Ma L, et al. Association between measurable residual disease in patients with intermediate-risk acute myeloid leukemia and first remission, treatment, and outcomes. *JAMA Netw Open.* 2021;4(7):e2115991. doi:10.1001/jamanetworkopen.2021.15991

eTable. Causes of Death Posttransplantation
eFigure. Forest Plots for Survival of MSD-SCT and AD-SCT

This supplementary material has been provided by the authors to give readers additional information about their work.

© 2021 Yu S et al. *JAMA Network Open.*

eTable. Causes of Death Posttransplantation

Cause of death	CMT group	auto-SCT group	allo-SCT group
	no. (%)	no. (%)	no. (%)
Relapse	58(89.2)	24(82.8)	28 (45.9)
GVHD-related			
aGVHD	1(1.5)*	0(0)	8(13.1)
cGVHD	0(0)	0(0)	5(8.2)
Treatment-related			
Infections	5 (7.7)	5 (17.2)	13 (21.3)
others	1(1.5)	0(0)	7(11.5)

Abbreviations: CMT, chemotherapy; auto-SCT, autologous stem cell transplantation; allo-SCT, allogeneic stem cell transplantation.

*Some patients received allo-SCT as salvage treatment in CMT group and one died of aGVHD

© 2021 Yu S et al. *JAMA Network Open.*

eFigure. Forest Plots for Survival of MSD-SCT and AD-SCT. (a) LFS. (b) OS. (c) GRFS

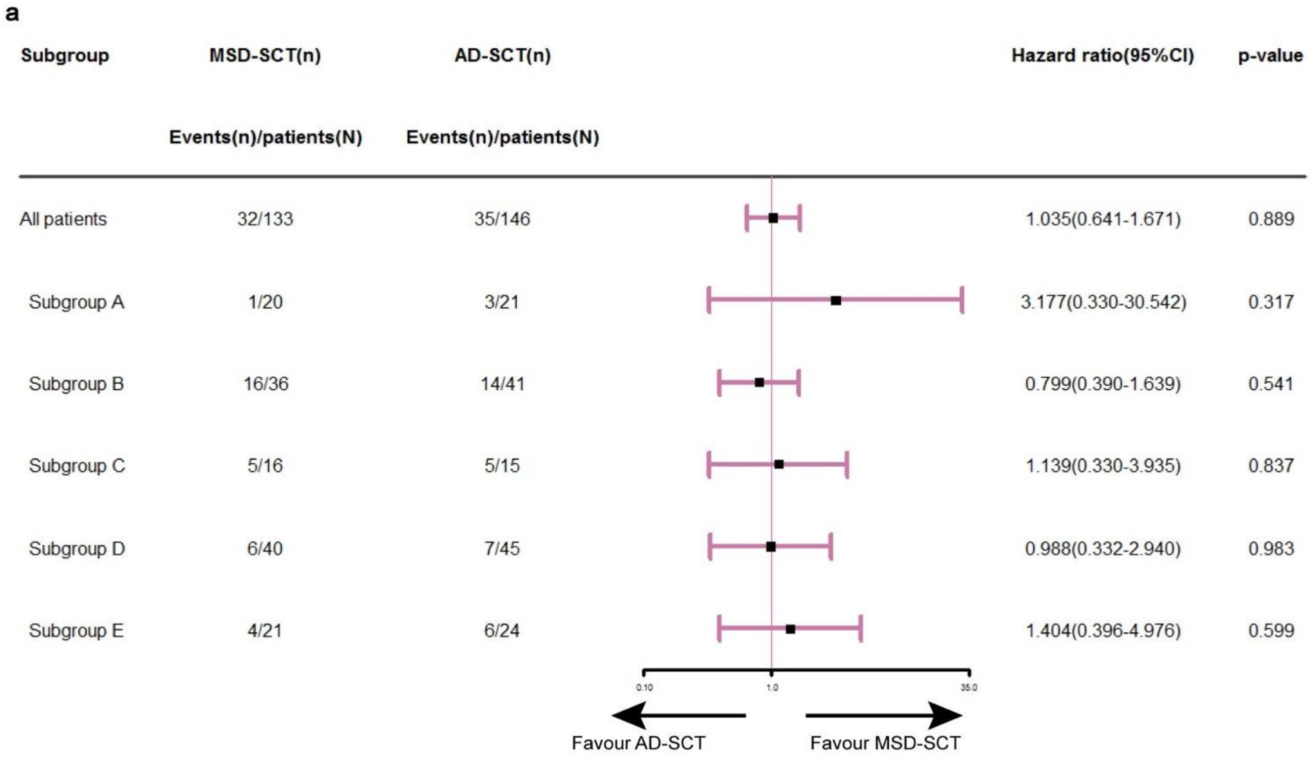

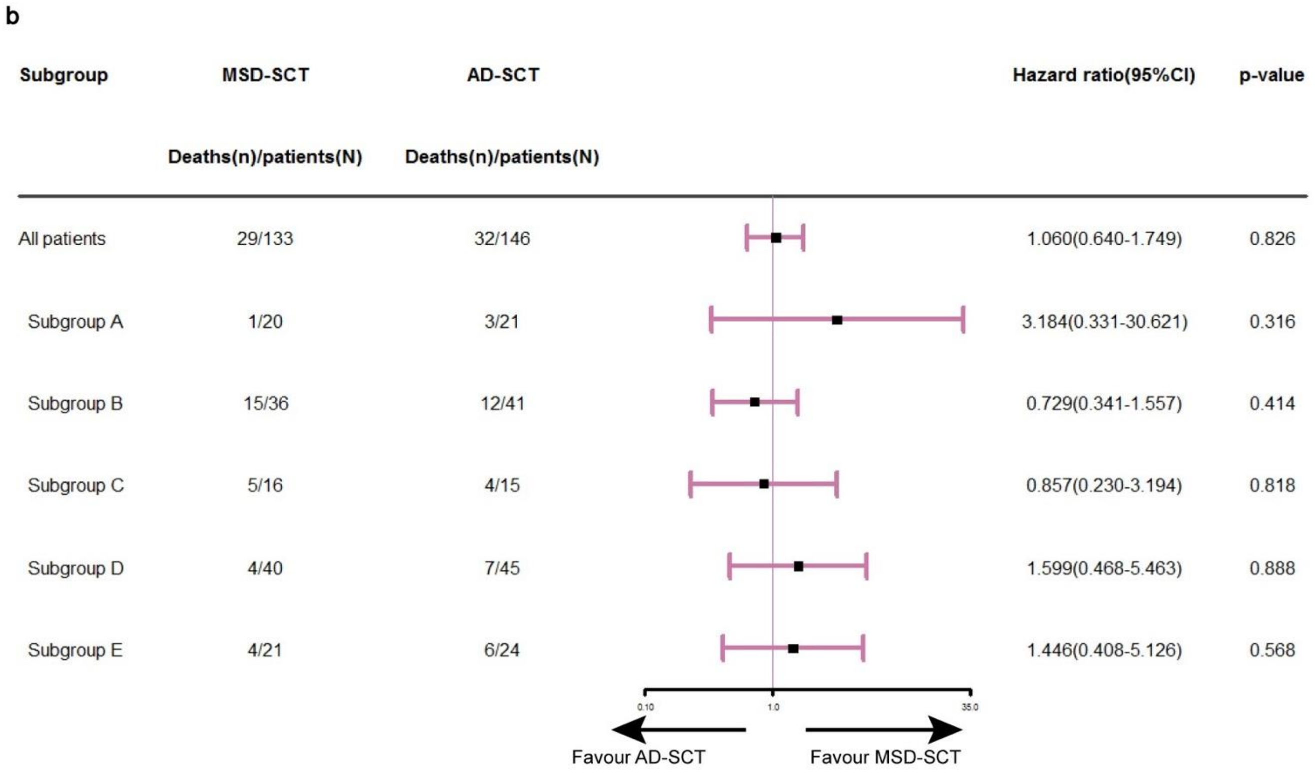

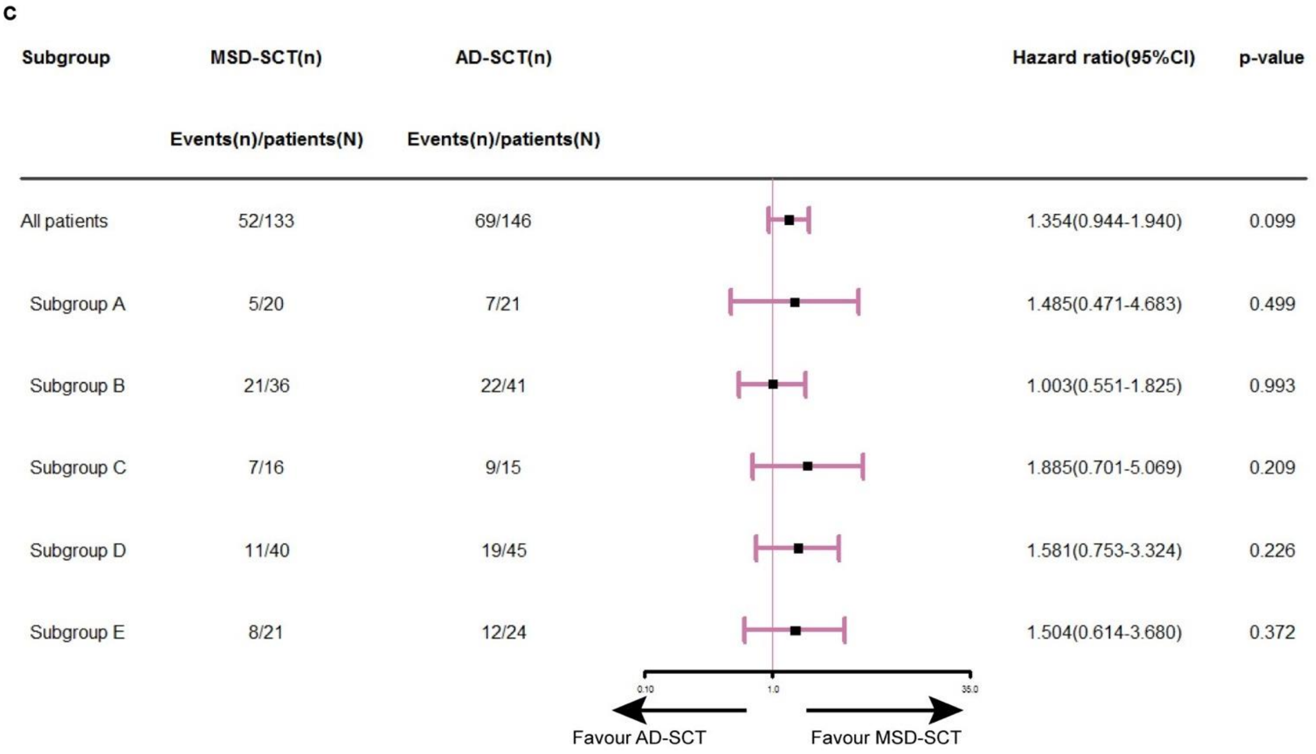

© 2021 Yu S et al. *JAMA Network Open.*

Abbreviations: MSD-SCT, matched sibling donor stem cell transplant; AD-SCT, alternative donor stem cell transplant.

Subgroup A, patients who become MRD negative after one course of chemotherapy and had persistently negative MRD; subgroup B, persistently positive MRD; subgroup C, recurrent MRD+ patients after being MRD-; subgroup D, patients who become MRD negative after two courses of chemotherapy; subgroup E, patients who become MRD negative after three courses of chemotherapy.

© 2021 Yu S et al. *JAMA Network Open.*
